# The roles of stakeholder experience and organizational learning in declining mass drug administration coverage for lymphatic filariasis in Port-au-Prince, Haiti: A case study

**DOI:** 10.1371/journal.pntd.0008318

**Published:** 2020-05-29

**Authors:** Breanna K. Wodnik, Didié Hérold Louis, Michel Joseph, Lee T. Wilkers, Susan D. Landskroener, Luccene Desir, Jean Frantz Lemoine, James V. Lavery

**Affiliations:** 1 Hubert Department of Global Health, Rollins School of Public Health, and Center for Ethics, Emory University, Atlanta, Georgia, United States of America; 2 National Ambulance Center, Ministry of Public Health and Population, Port-au-Prince, Haiti; 3 Radio Caraibes, Port-au-Prince, Haiti; 4 Hispaniola Health Initiative, The Carter Center, Port-au-Prince, Haiti; 5 National Programs for the Elimination of Malaria and Lymphatic Filariasis, Ministry of Public Health and Population, Port-au-Prince, Haiti; Elisabeth Bruyere Research Institute, CANADA

## Abstract

The World Health Organization (WHO) defines an effective round of mass drug administration (MDA) for lymphatic filariasis (LF) as one that reaches at least 65% of the target population. In its first round of MDA in 2011–2012, the National Program to Eliminate LF in Haiti achieved a 79% epidemiological coverage in urban Port-au-Prince. In 2013, coverage dropped below the WHO threshold and has declined year-over-year to a low of 41% in 2017. We conducted a retrospective qualitative case study to identify key factors behind the decline in coverage in Port-au-Prince and ways to address them. Our findings suggest that the main contributors to the decline in MDA coverage appear to be the absence of effective documentation of practices, reporting, analysis, and program quality improvement—i.e., learning mechanisms—within the program’s MDA design and implementation strategy. In addition to their contribution to the program’s failure to meet its coverage targets, these deficits have resulted in a high cost for the MDA campaign in both lost momentum and depleted morale. Through a proposed operating logic model, we explore how the pathway from program inputs to outcomes is influenced by a wide array of mediating factors, which shape potential participants’ experience of MDA and, in turn, influence their reasoning and decisions to take, or not take, the pills. Our model suggests that the decisions and behavior of individuals are a reflection of their overall experience of the program itself, mediated through a host of contextual factors, and not simply the expression of a fixed choice or preference. This holistic approach offers a novel and potentially valuable framing for the planning and evaluation of MDA strategies for LF and other diseases, and may be applicable in a variety of global health programs.

## Author summary

In order to eliminate lymphatic filariasis from a region, the World Health Organization recommends a strategy of preventative drugs delivered annually to the population. At least 65% of the population must participate for four to six consecutive years for this approach to be effective. In urban Port-au-Prince, Haiti, the number of people taking the drugs has been declining progressively below the 65% benchmark since 2012. We used a qualitative case study to identify key factors behind the decline in coverage in Port-au-Prince and ways to address them. We found that the lymphatic filariasis elimination program lacks the necessary design and program infrastructure to ensure reliable learning about problems faced by pill distribution teams on the ground, and the improvisations they introduced to address these challenges. The inability to incorporate these lessons into program improvements and refinements resulted in under-performance and contributed to the decline in coverage. Furthermore, the way that people experience the program is strongly influenced by a host of contextual factors, which shape potential participants’ experience of the program and, in turn, influence their reasoning and decisions to take, or not take, the pills.

## Introduction

Lymphatic filariasis (LF) is a parasitic neglected tropical disease (NTD) that may cause severe pain, disfigurement, and disability. Like many other NTDs, LF disproportionately affects some of the world’s most impoverished populations [1]. Those affected by LF often experience social stigma in addition to physical symptoms. Stigma contributes to weakened mental health and loss of social opportunities, all of which can intensify poverty [1]. The World Health Organization (WHO) recommends mass drug administration (MDA) as a preventative chemotherapy strategy in LF-endemic countries, and as the main strategy for the highly successful Global Programme to Eliminate Lymphatic Filaraisis, which it launched in 2000 [2]. In order to eliminate LF, MDA coverage, defined as the percentage of the population that have been treated with preventative chemotherapy, must consistently reach at least 65% in endemic areas for 4–6 years [3].

**Fig 1:**
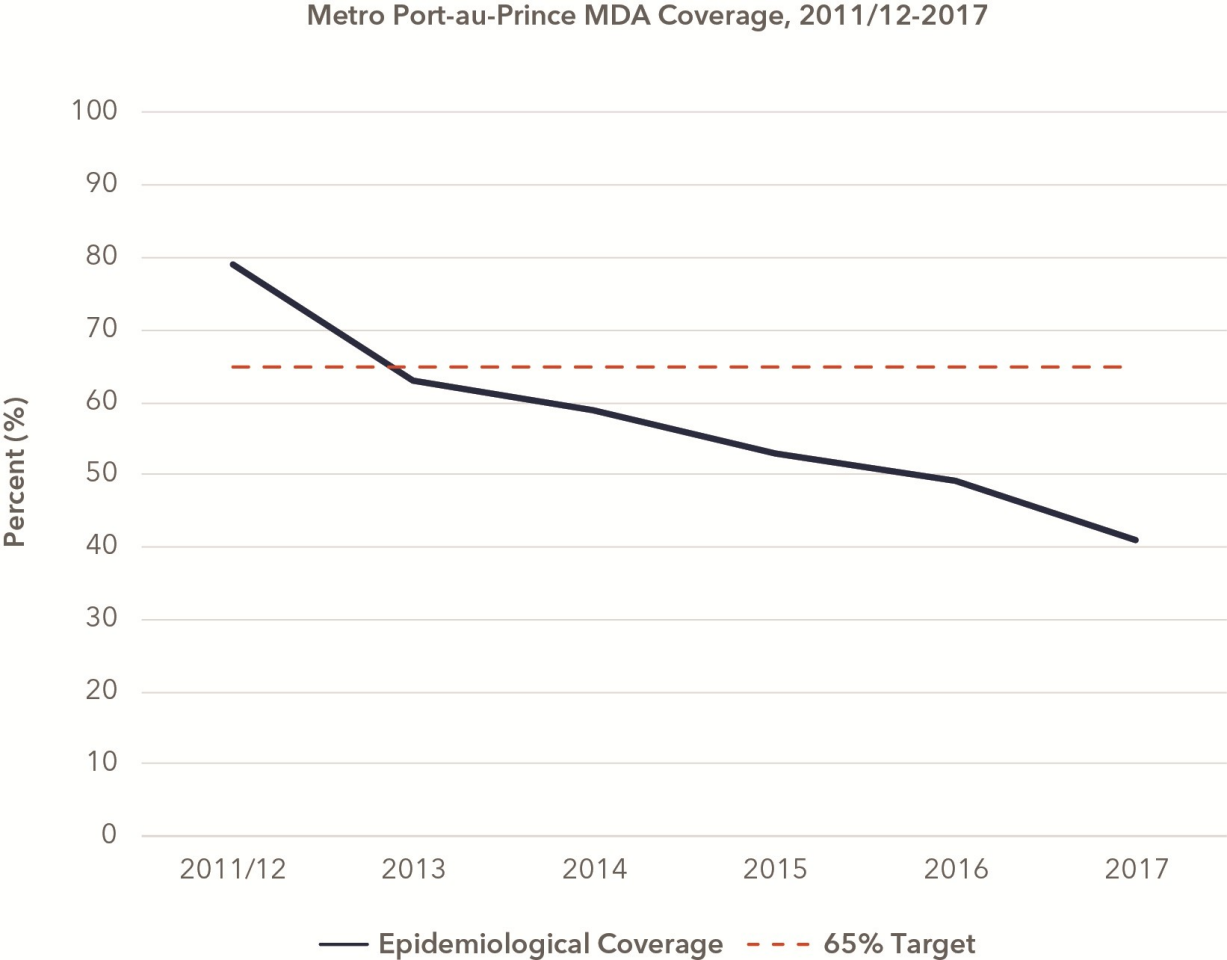
Declining epidemiological coverage from 2011/12 to 2017 in metropolitan Port-au-Prince, Haiti. All six communes in Port-au-Prince received MDA until 2016, after which one commune (Pétion-Ville) ended MDA due to sufficiently low LF antigen prevalence.

While LF is still endemic in 72 countries worldwide, Haiti is one of only four endemic countries in the Americas, and one of two still requiring MDA in the region [4]. LF affects both urban and rural populations in Haiti [5]. Following the launch of Haiti’s elimination program in Léogâne commune in 2000, annual MDA implementation in the metropolitan area of Port-au-Prince began in 2011 and 2012 with a chemoprophylaxis regimen of albendazole and diethylcarbamazine (DEC) [6]. The metropolitan area of Port-au-Prince consists of six communes (Port-au-Prince, Delmas, Carrefour, Cite Soleil, Pétion-Ville, and Tabarre), with a population estimated at just over 2.6 million as of 2015 [7]. Following a successful first round of MDA in 2011/12—i.e., epidemiological coverage of 79%–MDA coverage has been decreasing annually in the capital city, falling consistently below the 65% benchmark necessary for elimination (Fig 1). With the exception of Pétion-Ville commune, which stopped annual MDA following the 2016 distribution due to a sufficiently low LF antigen prevalence in the population, the five remaining communes making up metropolitan Port-au-Prince continue MDA today.

The problem of persistent LF in an urban area after many years of MDA is not unique to Haiti [8–14]. Urban areas have been particularly challenging environments for MDA programming for LF and other diseases preventable through chemoprophylaxis [11–14]. Noted challenges for MDA programs in urban areas include accessibility (e.g., unwillingness of drug distributors to enter high-income residential areas, or difficulties posed by working in high-rise apartment complexes), personal security risks for drug distribution team members in high-crime slum settlements due to gang-related violence and intimidation, high rates of population mobility and migration, and fragile trust, both in people and institutions, which is “difficult to build and can be quick to erode” [14].

**Fig 2:**
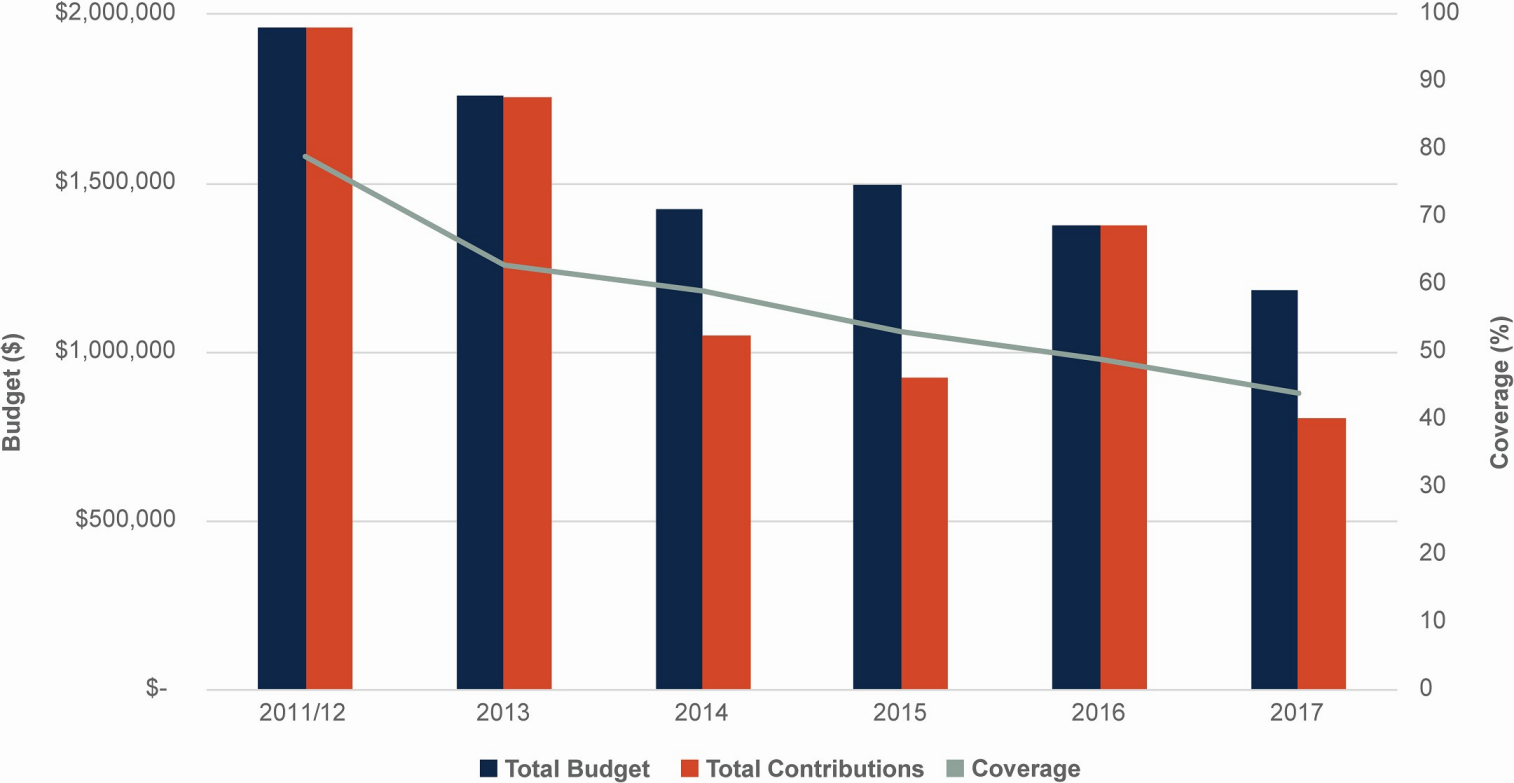
The relationship between annual budget and total contributions for MDA in Port-au-Prince, Haiti, and LF MDA coverage in Port-au-Prince. All six communes in Port-au-Prince received MDA until 2016, after which one commune (Pétion-Ville) ended MDA due to sufficiently low LF antigen prevalence. Data provided by the Haiti Ministry of Health.

In addition to these common urban challenges, MDA implementation for LF in Port-au-Prince has faced a great number of political, physical, and social challenges, including political crises and instability resulting from the 2004 coup d’état, a devastating earthquake and subsequent cholera outbreak in 2010, and the 2016 strike from Hurricane Matthew [6]. Funding challenges have also plagued the program since its inception in the metro area, with the declines in program funding closely tracking the declines in MDA coverage (Fig 2).

Against this background, we conducted a retrospective qualitative case study to identify potential contributing factors to the low MDA coverage for LF in metro Port-au-Prince. We used a community and stakeholder engagement (CSE) framework to guide both the study design and analysis [15]. Our findings offer a novel and potentially valuable framing for the planning and evaluation of MDA strategies for LF and other diseases, and may be applicable in a variety of global health programs.

## Methods

### Overview

This study employed a retrospective qualitative case study approach, informed by a literature review and an original CSE framework developed by one of us (JVL) through a series of empirical case studies [15], to identify key factors behind the year-over-year decline in coverage in Port-au-Prince and ways to address them. The study involved in-depth interviews with a sample of MDA drug distribution team members, program management staff, individuals who have agreed to take drugs in previous MDA campaigns, and those who have not.

### Literature review

Papers considered in the literature review of existing research fell into five main categories; 1) LF MDA specific to Haiti; 2) LF MDA in contexts other than Haiti; 3) LF MDA practices specific to urban contexts; 4) relevant MDA practices for diseases other than LF; and 5) LF papers that were not specifically related to MDA. A total of 44 papers were identified as relevant for review.

The aim of the literature review was to establish a preliminary account of how obstacles to effective MDA campaigns for LF—and MDA campaigns in general—are described in the literature and to compare these with the CSE framework [15] that provided the conceptual foundation for this study. Each of the 44 papers was analyzed and coded according to grounded theory data analysis procedures [16]. The resulting analytic codes were integrated into a ‘network view’ in ATLAS.ti (version 8.3.0), which provided an initial visual representation of the existing literature and was used to guide the development of the qualitative case study interview strategy and train the data collection team. The formal analysis of the literature contributed to what Corbin and Strauss refer to as “theoretical sensitivity”, i.e., the extent to which the analysts are sensitized to key concepts and ideas related to the phenomenon under investigation [17].

### Retrospective qualitative case study

This study employed a retrospective qualitative case study approach using grounded theory data collection and analysis methods [16], which our team has used successfully in several other CSE case studies [18–21].

#### Sampling strategy

**Fig 3:**
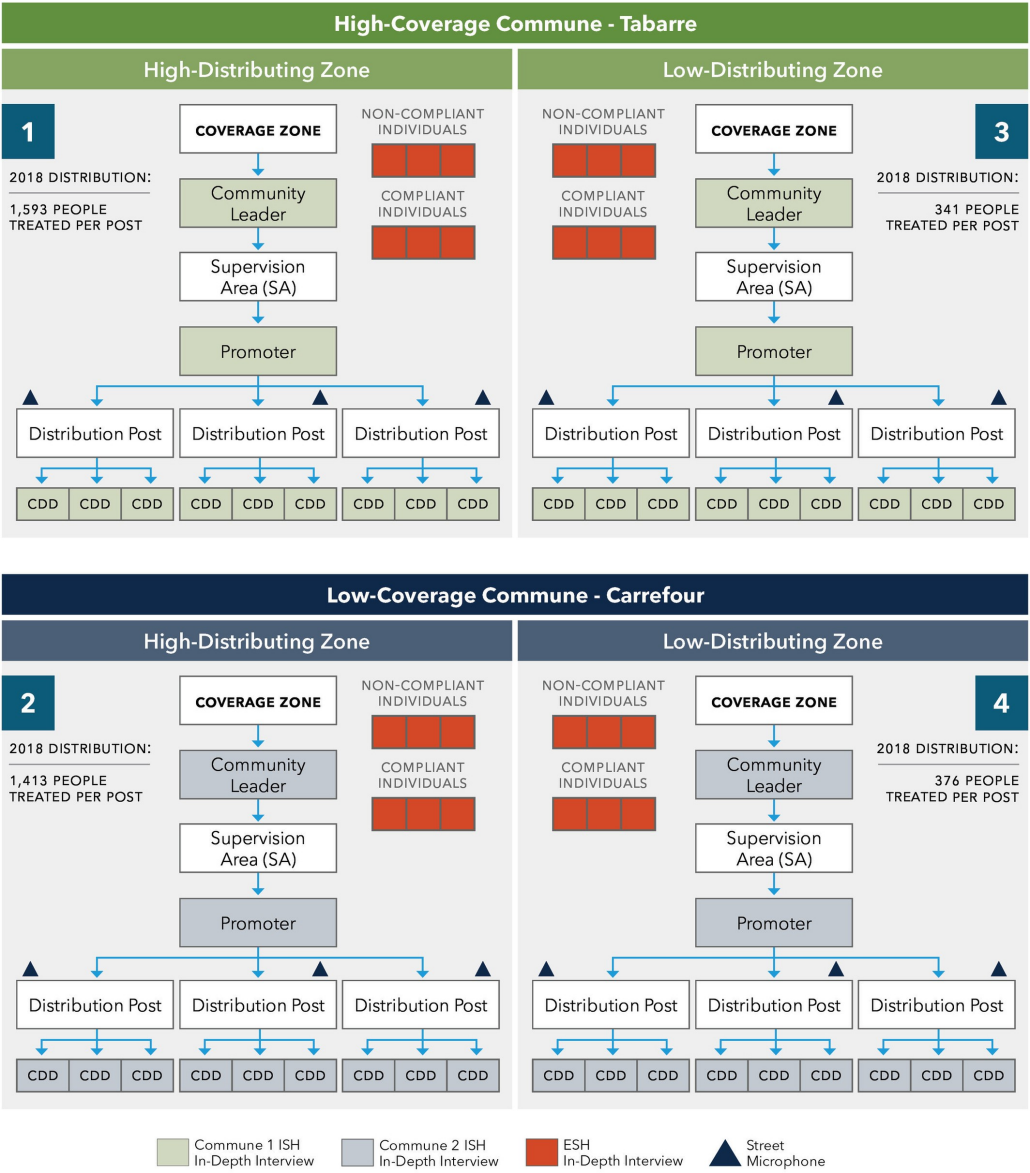
Sampling strategy for qualitative interviews.

The sampling strategy for the qualitative case study was developed to explore the working relationships between the drug distribution teams, which are organized in a cascade of supervision from community leaders, to their respective promoters, and the community drug distributors (CDDs) who manage the distribution posts under the promoters’ supervision. We conducted interviews with both internal stakeholders (e.g., leaders, promoters, and CDDs; n = 31), and external stakeholders (e.g., compliant and non-compliant community members; n = 44) at one high-distributing zone and one low-distributing zone in each of two communes, Tabarre and Carrefour (Fig 3). All participants were 18 years or older, and provided written informed consent.

The epidemiological coverage (e.g., population treated divided by the total population) from each MDA year was averaged to identify the most consistently high-coverage and low-coverage communes of the five communes still participating in MDA in metropolitan Port-au-Prince (the sixth commune, Pétion-Ville, ended annual MDA in 2016). Using data from 2011/12 through 2018, Tabarre and Carrefour communes were identified as having the most consistently high epidemiological coverage (90.0%) and low epidemiological coverage (54.4%), respectively.

Denominator data for each drug distribution post were not available, though posts were expected to serve approximately 1,000 people each [22]. Since true epidemiological coverage could not be calculated, zones were labeled as high-distributing or low-distributing based on the number of people treated at the posts in those zones in 2018. That year, the median number of people treated per post in Tabarre was 807 (range: 250–1,726), and the median number of people treated per post in Carrefour was 819 (range: 140–2,579). Fig 3 lists the average number of people treated by the three selected distribution posts for a selected zone.

#### In-Depth key informant interviews

The key informant interviews were conducted, primarily with internal stakeholders. These interviews covered issues such as their views on the LF MDA campaign design, the specific activities and practices they employed in their work and their perceived outcomes, challenges they faced in the implementation of the LF MDA campaign, and insights about how they were received by members of the public during the drug distribution process. Key informant interviews with external stakeholders elicited their perceptions of the program and the LF MDA campaigns, their personal experiences with LF MDA compliance or non-compliance, and their views about facilitators and barriers to LF MDA compliance for themselves and others.

In keeping with grounded theory methods, interviews were structured to elicit interviewees’ perspectives and experiences, in their own words and framing, rather than requiring them to respond to questions from an interview guide set from the interviewer’s perspective. The interviewer ensured that the key concepts in the CSE conceptual framework [15], and issues that arose in the literature review, were adequately explored once the interviewees had related their own experiences and perspectives. The CSE conceptual framework outlines key elements of the foundations, planning, design, management, and evaluation phases of a project or program (e.g., enabling conditions for CSE created by the funding agreement, the integration of insights from CSE into the day-to-day management of the program) that, if better understood, could contribute to a strengthened evidence base for CSE and lend critical insights to both funders and practitioners about how CSE functions in various contexts [15]. Data analysis was conducted between sets of interviews for each sample area, and any new insights identified during the analysis were explored with successive interviewees [16].

Interviews were conducted in Haitian Creole at a private location determined by the interviewer and interviewee. The interviews, conducted between September and December of 2018, ranged in length from 10 to 60 minutes and were audio recorded, transcribed verbatim, and translated into English for analysis. With some exceptions, all interviews were completed in one zone before moving on to the next, and zones were visited in the order outlined in Fig 3; within each zone, the community leader and promoter were interviewed, and CDDs and external stakeholders interviewed until saturation was reached.

#### Street microphone interviews

Given the design and primary aims of the study, there was no natural sampling frame from which to draw a sample of external stakeholders to gauge their attitudes about, and experiences with, the MDA campaigns in Port-au-Prince. Although the sampling for the in-depth key informant interviews included several compliant and non-compliant individuals who were identified by internal stakeholders in each zone (Fig 3), we decided to complement these interviews with an additional convenience sample of the general public. We aimed to create a rough approximation of how CDDs would encounter passers-by on the street during drug distribution days by positioning an interviewer at nine of the twelve selected distribution posts (posts in Zone 4 were inaccessible for these interviews due to its remoteness, mountainous topography, and civil unrest). These ‘street microphone’ interviews were conducted at the distribution post site by a local journalist who sought consent from passers-by to ask them whether they had ever taken the MDA drugs in previous campaigns or not, the reasons for their decision, and their general thoughts or observations about the program. These interviews were approximately 4 to 6 minutes in length.

### Analysis

We aimed to produce an explanatory account combining detailed description of the social processes involved in the planning, design, and management of the MDA campaigns, with explanations of how various factors contributed to the declines in coverage [16,19,23,24]. Grounded theory was selected as a foundation for the analytical methodology as it is a theory-generating approach, which emphasizes the experience of participants and their interpretation of those experiences, rather than an approach which seeks the confirmation of the research team’s hypotheses [16,19,24].

We coded the written transcripts using ATLAS.ti software to identify patterns, emerging categories and key concepts [16,17,23,25]. Audio recordings from the street microphone interviews were analyzed directly in Haitian Creole, and insights discussed with the analysis team. In keeping with the grounded theory method, the analysis team compared findings within and across interviews and among categories using a constant comparative approach [16,17]. Information considered in the development of the final set of recommendations included all coded transcripts and related analytic memos, audio street microphone interviews, findings from the literature review, relevant epidemiological data, and meeting notes from weekly debriefing and analysis sessions.

### Ethics statement

This research was reviewed and approved by the Emory Institutional Review Board (Atlanta, Georgia, USA; Study ID IRB00105324), and by the Comité National de Bioéthique of the Haitian Ministry of Health (Port-au-Prince, Haiti; Study ID 1718–80). Written informed consent was obtained from all participants; all participants were 18 years or older.

## Results

### Participants and distribution post sites

Participants were recruited from two of the six communes in metropolitan Port-au-Prince, Tabarre and Carrefour. Tabarre is a small commune located in the northeastern part of the city. Carrefour, considerably larger than Tabarre, sits at the southern borders of Port-au-Prince and contains extremely mountainous terrain, some of which is reportedly only accessible by foot or mule. Although a population-wide census of Haiti has not been conducted since 2003, the current populations of these communes are estimated to be 130,283 and 511,345, respectively [7].

We conducted a total of 41 in-depth interviews (internal and external stakeholders) and 34 street microphone interviews (external stakeholders). The sampling strategy was developed with the aim of identifying differences between high-distributing and low-distributing zones, which we hoped would deliver further insights into more- and less-effective distribution strategies. However, we did not see consistent differences between the four zones in distribution approach, common attitudes, or other factors. Internal stakeholders in all four zones discussed similar programmatic strengths and challenges. The absence of anticipated differences was significant because it suggested that the strong emphasis on “systematic non-compliance” among prospective MDA participants in the literature [26], and among program partners, might have obscured systemic features of the program itself, which might account for some of the observed decline in compliance.

Below, we present two levels of findings: a general explanatory model or ‘operating logic’ of the MDA programming, i.e., the pathways and mechanisms that link the program elements and coverage decline; and five broad and highly inter-related programmatic factors that we have identified as likely contributors to the decline in MDA coverage.

### Operating logic of the LF MDA program

Potential participants’ exposure to the LF MDA program is determined both by program inputs and by a wide array of mediating factors, which shape their experience of the program and in turn, influence their reasoning and decisions to take, or not take, the pills.

**Fig 4:**
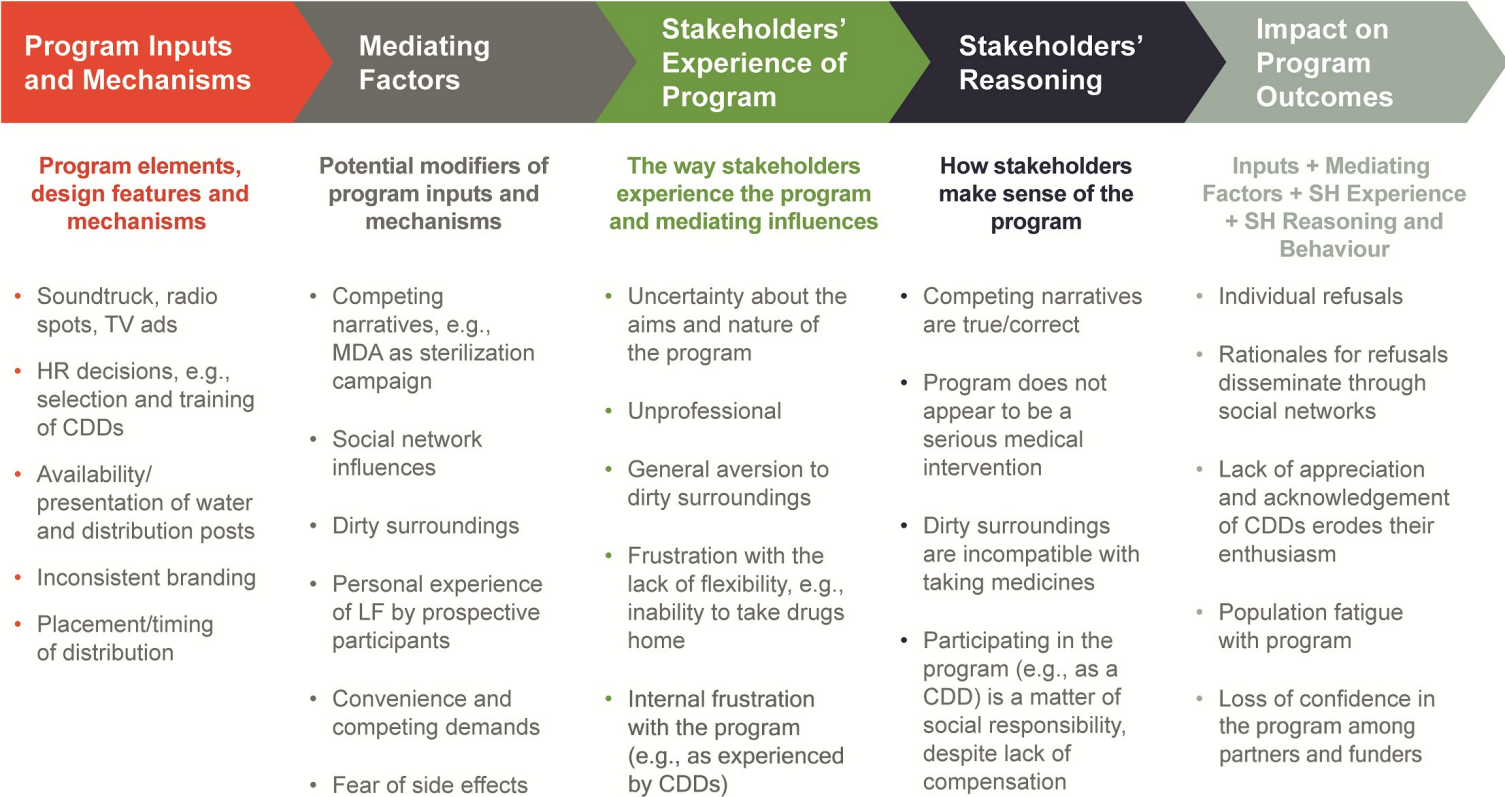
Operating logic relating program inputs to ultimate program outcomes.

Fig 4 provides an overview of this operating logic of the program, which arose from our findings. The LF MDA program is made up of a wide range of elements including design features, such as the placement and timing of drug distribution, and various actions, decisions, and practices that serve as the mechanisms through which the program is operationalized. These program inputs are introduced against a complex backdrop of contextual factors. Some of these factors can have direct, or indirect, effects on the ways that various stakeholders experience the MDA campaign. For example, some of the distribution sites are in parts of the city where there is garbage or generally dirty surroundings. This specific feature of the context within which the program is being implemented was viewed by some stakeholders as incongruous with a program that requires someone to take a medicine. These stakeholders experienced the overall program in a negative way as a result of the dirty surroundings, and because the setting was not conducive to taking medication, they ultimately refused to take the drugs. Some of the unique features of urban environments have compounded these challenges and have made it difficult to determine the best LF MDA program design for urban areas. It is very likely that the socio-political and economic circumstances in Port-au-Prince, combined with its unique urban geography, present a particularly extreme set of these *mediating factors*.

This representation of our findings offers a novel way to understand the problem of non-compliance with the LF MDA program. It moves away from the singular focus on rationales that prospective participants hold independently (i.e., specific grounds for refusal) and puts these rationales and behaviors (i.e., decisions to either refuse to take drugs or comply) in the broader context of the way these stakeholders experience the program itself and the way these experiences are mediated by other factors at work in the context. For example, in Port-au-Prince, public communications about the MDA campaign must compete with prevalent narratives about sterilization, or exaggerated rates of side effects. As well, the most diligent, knowledgeable and personable CDDs may not be able to convince some people to take the drugs without the supervision of a doctor. Even the most dedicated community leader cannot effectively coordinate such a large-scale operation with limited phone card funds or budgets for transportation of materials. The best prepared drug distribution post might be unable to encourage some people to take the drugs if it is located in an area surrounded by trash. And the best prepared drug distribution post might be unable to encourage some people to take the drugs if they have no personal experience with, or perceived risk of, LF. But against this general program operating logic, we also identified five specific features of the program that appear likely to contribute to the decline in MDA coverage.

### Five programmatic factors likely contributing to the decline in MDA coverage

#### 1. The design and implementation of the program's MDA campaigns do not adequately prioritize the convenience and acceptability for prospective participants

WHO guidance for the design and implementation of MDA programs for LF focuses primarily on epidemiological considerations, with little explicit attention to programmatic details [27]. For example, whereas drug procurement and supply chain issues are addressed in some detail, there is no explicit guidance on how to plan and manage supply chains for clean water for the distribution posts. And although there is considerable attention given to the crafting and delivery of key messages for program communications, there is no guidance on how to establish branding and strategic partnership practices that reliably communicate and reinforce the program’s medical and public health mission. Yet, it appears that details like these are precisely the ones that have the most direct impact on shaping prospective participants’ experience of the LF MDA program, and, by extension, their willingness to comply.

*“Some people don’t want to take it because they are at work*. *While we are talking about work*, *I was wondering if it would be possible to have a post at some of these work sites because some people at the times*, *they are working they can’t find someone in their area to take it*. *If they could get it at their work*, *it would also be better because I’ve been able to get multiple people that way*. *They tell me that when they are at work*, *they can’t find someone to give it to them*. *Those are some solutions to get some people to give it to some people at their jobs*. *Because once people start working you can’t find them anymore*. *It can be 4*, *5 o’clock and they’re still at work*.”—Community Drug Distributor

#### 2. The program does not have the necessary design or capacity to “learn-by-doing”

The WHO’s 2000 guideline for program managers of national LF elimination programs states that “(t)he manual will be adapted and changed as the LF community “learns-by-doing”” [27]. Our findings from drug distribution team members suggest that there are no established feedback channels or reporting procedures by which they can document their experiences of logistical challenges—e.g., the inability to provide food, or challenges with the transport of water—and insights about the way prospective participants perceive the program. Instead, the program appears to function as an open system, in which the drug distribution teams exercise their own judgement and improvise creative solutions to problems as they arise—e.g., purchasing or negotiating donations of food, loans of chairs for the distribution posts, or leaving their distribution posts to seek willing participants in their neighborhoods during low traffic periods. But because there is no reliable way to track these improvisations or their impact, or to document the problems that necessitated them, the program lacks the necessary capacity to “learn-by-doing”.

“*So*, *we would go to them at 10 a*.*m*. *and bring it to them at the schools*. *Even then*, *we have to carry everything*. *But I am wondering if they can do it another way*, *like make a group of people go to the school and a group go to the community*… *when there’s school*, *we go to the school first at the time the students have already eaten at the school*, *then we go back to the post*. *Sometimes this poses an issue because when they come to check the post*, *they might not find us and our supervisor wouldn’t be too happy about that*. *But that’s how we do it because we want to make sure that most people take the medication*.*”—*Community Drug Distributor

“… *compared to how we see the field*, *everything we tell them every improvement that has to be made*, *they say they’ll do it but they don’t do anything*.*”—*Community Leader

#### 3. The design and implementation of potentially important program mechanisms are constrained, or precluded, by a lack of funding and/or logistical support

The lack of attention to learning, described above, may reflect—at least to some degree—the seemingly negligible significance of many of the challenges reported to us by drug distribution team members. The program operates under a set of design assumptions, which are neither accounted for monetarily nor in the MDA country protocol. These details may seem small individually, but compound one another and affect the performance of the program and the way that individuals experience it.

“… *if he takes 10 bags of water he is forced to rent a car*, *what they give [the stipend] can’t respond to this*. *What they give it doesn’t respond every day for everyone when they have to climb when there isn’t water for people*.*”*—Promoter

“… *for the zones that are up the mountain*… *sometimes we go on mule and I pay [for] the mule 200 or 300 gourdes*.*”—*Community Leader

“… *the program used to give me 200 gourdes for phone cards to put on my phone for the year*… *so I use the money from my wife’s business to do the job*… *and I also use other money from work in the province*…*”*—Community Leader

#### 4. The commitment and motivation of drug distribution teams is nurtured by their personal values and determination, not by the program

Nearly every promoter and community leader mentioned having to use their own money or borrow from friends and family in order to fund transportation for themselves and the CDDs and to purchase supplies, such as food for CDDs on distribution days and snacks as incentives for community members to take the pills. One CDD said of his promoter that “he is the only one who will ask us how are we doing, if we have eaten, if we had something to drink, he is the only person to do so”.

Payment and stipends to CDDs in both Tabarre and Carrefour communes was viewed as insufficient, poorly timed, late, or absent entirely. Underfunding or poor timing of the stipend has direct influence on the morale, and likely also the productivity, of CDDs [28]. But despite the challenges, they remain remarkably dedicated to their work. The wholesale reliance on their on-going cooperation creates vulnerability in the current program design.

“*I have a strong devotion to this work*. *My group has three people and we all have a strong devotion to the work that we do*. *We collaborate amongst each other*, *we take the bag of water*, *the equipment that we have if they are all ready*, *and we organize ourselves so that we can do the distribution*. *We have a strong desire and volition to do this*.*”—*Community Drug Distributor

#### 5. The genesis, governance, and ultimate goals of the program remain unclear to many Haitians and this results in persistent challenges for its credibility and trustworthiness

The program lacks credibility among many of the internal stakeholders (e.g., drug distribution teams) and external stakeholders (e.g., community members) we interviewed. This credibility deficit appears to arise from a lack of clarity about the identity of the program, i.e., its genesis, funding, supporting partnerships, aims, and value for Haitians, and is exacerbated by inconsistent branding and by its vulnerability to competing narratives, such as rumors that the government is using the program as a way to sterilize the population.

Perhaps most importantly, the credibility of the program is undermined by the significant disconnect between the nature of the program—a medical and public health intervention—and its perception by many in the public—and even by some members of the drug distribution teams—as unprofessional and half-hearted.

“*Choose a place that is favorable to give to them because it’s a medicine against a disease*, *you’ll choose a place that’s cleaner so people can have confidence to take it once the people arrive to a place that’s clean*, *it’s medicine they are giving*, *this could encourage them to take it*, *but if they find people sitting anywhere*, *people won’t give it any importance because of the way they are giving the medicine*.*”—*Compliant Community Member

“*The medicine gives you gwogrenn (hydrocele)*. *It hasn’t happened to me personally*, *but I know it’s happened to a lot of people*. *That’s one reason why I don’t take them*. *A lot of people don’t take the pills for this reason*. *It’s not that they are difficult to find–I choose not to take them*. *The people are easy to see*, *with their t-shirts*. *I don’t want to take medicine from these people with t-shirts*. *If a doctor told me I needed this medicine for a disease*, *then I would take it*.*”—*Non-compliant Community Member

### Limitations

Data collection for the study was suspended on several occasions due to demonstrations and civil unrest in Port-au-Prince during critical data collection periods. These circumstances prevented travel, and intermittent phone and internet access during that time made communication impossible at times, hindering data collection and analysis.

High- and low-coverage zones were defined by the number of pills distributed, as opposed to the percentage of a zone’s population that complied with MDA, due to an absence of denominator data. Distribution posts should, by theory, serve approximately 1,000 people each, but because of issues like daily commuting and the presence of large participating schools in some zones, the labels of high- or low-coverage may not be accurate.

As a result of these limitations, we were unable to generate sufficient data to support specific zone-by-zone comparisons, as we had anticipated in our sampling strategy. We could not, with confidence, attribute differences between high-coverage and low-coverage areas to leadership, promotion, or day-of-distribution activities specific to those sites. By selecting communes and zones based on their reported rates of coverage and distribution, we hoped to increase the likelihood that we would be able to identify good practices by the drug distribution teams that might have contributed to high distribution and to compare them with the practices associated with low distribution outcomes. And we hoped to see any differences in drug distribution practices in each zone reflected in the attitudes and experiences of members of the general public (external stakeholders) in those zones who had either agreed to take the drugs in a previous round of MDA, or who had not agreed to take the drugs. Instead, we have been limited to providing broader, programmatic analyses that we believe to be broadly applicable to all drug distribution efforts in Port-au-Prince.

## Discussion

Evidence throughout the literature on LF MDA demonstrates that issues of non-compliance have plagued countries all over the world [6,8,26,29,30]. Specific rationales for non-compliance with MDA drug regimens have been widely reported in previous studies, and many or most of these rationales have been echoed by our study interviewees. These include, but are not limited to: fears of “government” attempts to sterilize the population; fear and avoidance of side effects that either make people sick and/or mimic symptoms of the disease; the program’s lack of medical or public health credibility; rejection of Directly Observed Treatment approaches, either for feelings of imposition or because of the lack of availability of food to take with the pills at distribution sites; and a lack of understanding about the rationale for the MDA strategy.

Two observations are relevant here. First, our study was not designed to produce a survey of the rates and distribution of these rationales among non-compliant populations in metro Port-au-Prince. Second, such a study would be complex and expensive. More importantly, it is unclear how such a study would direct the actions necessary to improve the program’s performance. For example, we encountered people who took the drugs, but related similar concerns about the program as other people who did not take the drugs.

“… *when people go to [location of a drug distribution point]*… *they are mobilizing people to come take the medicine*… *[But] normally this place is very dirty*, *it became like a ravine*, *in this moment it’s where people drop all of their garbage and I see that it’s not good*, *and then it’s on the floor that they put the bags of water*, *this makes me not want to take the medicine in these places*.*”*—Compliant Community Member

“*In Haiti*, *I do not like the way the people give out the medication*. *That’s why I don’t even like to take it like that*… *when the people are sitting in the street to give out the medication*, *I don’t trust them*.*”*—Non-compliant Community Member

“*Yes yes*. *I am not saying that I am scared of it*… *it’s the way they are distributing it in the street*. *If the center was stable where they are giving it out*, *whenever I feel something I would just go and say that I am going to this center*.*”—*Non-compliant Community Member

As well, a survey of motivating rationales is typically cross-sectional and retrospective in nature, which can only treat the motivating rationales as fixed personal traits, independent of the influence of contextual factors. But this is a gross simplification of the social and psychological complexity of human decision-making. As a point of comparison, similar challenges have been addressed in other sectors, in particular in marketing and consumer purchasing behavior. Some of the key lessons from the academic business management fields provide an instructive analogy for understanding and addressing the challenges facing the LF MDA program outlined above.

The first lesson is that consumers’ preferences, choices and brand-loyalty are powerfully influenced, not only by advertising, but also by their overall experience at the point of sale [31,32] The second lesson is that consumer preferences are fragile and vulnerable to influences beyond the control of the companies that are producing products and advertising campaigns, such as the social networks that consumers inhabit and factors that can limit access to the products, or advertising, such as access to technology, physical geography, access to transportation, and limits to personal mobility [33]. The third lesson is that once preferences have been established, brand-loyalty is driven primarily by on-going experiences of value. Consumers will generally tolerate some challenges to their brand-loyalty—e.g., competing advertising, or negative attitudes within their social networks, and even some limitations on accessibility—as long as they continue to experience value in their purchases [34]. The fourth lesson is that significant investment is required by companies to allow them to continuously learn about consumer decisions and the factors that shape them. The explosive growth of online sales platforms such as Amazon are due overwhelmingly to the ability to collect and analyze enormous amounts of data about precisely these issues [35]. And the final lesson is that companies must invest in the creation and maintenance of the technical and human infrastructure, including management systems, necessary to translate these lessons into on-going improvement and refinements to their strategies in order to improve or protect their end goals, e.g., sales [36]. It is very likely that some of the seemingly trivial program details reflected in MDA funding, such as ensuring clean and clearly-branded distribution points and the availability and reliable delivery of clean water, will be the first to be cut when “program efficiencies” are sought. But our findings suggest that these may be critical factors in the decision-making of prospective participants. It is perhaps not surprising, then, that declining coverage closely corresponds to a mirrored decline in annual budget for the Port-au-Prince LF MDA program (Fig 2). Addressing this challenge effectively is likely to become even more important as LF programming shifts towards endgame strategies, where rising costs for transmission assessment surveys and post-MDA surveillance will have to compete with funds for ongoing MDA [37].

These lessons provide a broader framing for our findings. They reinforce our analysis that the decisions and behavior of individuals—in this case the decision whether or not to take drugs in LF MDA campaigns—are a reflection of their overall experience of the program itself, mediated through a host of contextual factors, and not simply the expression of a fixed choice or preference. The lessons from consumer psychology also help us understand that these decisions are driven largely by perceptions of value. This shifts the burden from the prospective MDA participant to “make the right decision” to the program, to ensure that the prospective participant has a clear value proposition motivating their decision.

It is a much simpler task to advertise the valuable features of a new mobile phone, than it is to explain the purpose, logic, and potential value of LF elimination, and MDA as the main tool to achieve it. But without a clearer and more persuasive account of the value of the MDA campaigns, many of the other competing influences described in our findings are likely to prevail and limit participation.

Perhaps the most relevant and important lesson from the consumer psychology literature is the central importance of investment in learning strategies as the cornerstone of success in consumer markets. Successful companies make extraordinary investments in learning about consumer behavior in order to win customers and lock down their brand loyalty. These investments are in the form of a variety of research strategies—e.g., experimental economics, eye-tracking studies of customer attention, and on-line purchasing behavior patterns. But more importantly, they come in the form of the human and technological infrastructure required to translate the findings of these studies into effective management and implementation strategies [36]. The main “competitors” for the LF MDA program are the many mediating contextual influences that we have described in our findings, and the many shortcomings and vulnerabilities in the program’s current design that are destined to continue without the necessary investment in learning and the corresponding improvements in program implementation.

The lack of reliable learning strategies in the LF MDA program design has four main consequences. First, it means that there is no single accurate account of the program, as it is actually delivered, and therefore no reliable way to build a diagnostic evaluation of declines in the program’s performance. Second, it results in discouragement and, in some cases, disillusionment among some of the drug distribution teams, who are trying to make the program work by identifying and responding to challenges as they arise through the course of drug distribution and who may ultimately come to see these efforts as futile. Third, the inability to identify and address aspects of the program and contextual factors that have had a negative influence on prospective participants’ experience of the program, e.g., its perceived lack of professionalism, means that these factors are almost inevitably repeated in each successive round of MDA rather than being eliminated through specific changes to program design. And fourth, the lack of evidence that results from the absence of learning mechanisms compounds the already considerable challenge of evaluating such a complex program and represents a significant obstacle to meaningful accountability for funders and partners, and, more importantly, to the public, especially those Haitians who have taken the drugs over and over again through the multiple rounds of MDA.

## Summary of findings and recommendations

There are no obvious indications that the experiences of potential participants have been anticipated in the implementation plan or in the underlying theory of the LF MDA program in Port-au-Prince. There are no clear learning mechanisms built into the day-to-day operations of the program. As a result, there is no way to learn about how the program is operating in the various contexts of metro Port-au-Prince, and no way to feed that information back to inform program design and management. And, even if learning feedback loops did exist, it seems likely that the current program design and management structures would be inadequate to allow meaningful responses to the lessons learned. These are challenges for program management, but they almost certainly also reflect the progressive decline in the program’s budget, which necessitates a wide range of trade-offs and compromises, some of which almost certainly correspond to the factors described, above. The program lacks credibility with both the public and the drug distribution teams, and there is a general feeling from those teams that the forward momentum for the program comes from the bottom up (i.e., from the drug distribution teams to the program management), rather than from the top down, despite the challenges reported, above. We expect that these broad programmatic challenges are present in many MDA strategies for LF and other diseases and may therefore represent a significant opportunity to improve the performance of a wide range of global health programs.

In light of these findings, we are currently working to implement the following recommendations:

- Revise the program’s implementation guide to address details of program delivery that are most likely to negatively affect prospective participants’ experience of the program.
- Establish the necessary documentation procedures and feedback channels for the drug distribution teams to help them capture important lessons about how the program is working—to enable the program to “learn-by-doing”—and to inform any necessary adjustments to the program’s design or management.
- Prepare an all-cost budget that addresses on-going logistical challenges for drug distribution teams (e.g., the transport of water to distribution posts) and explicit budget justifications for these projected costs. Use these revised budgets to facilitate dialogue and deliberations among funding partners about the feasibility of increasing budget allocations to more effectively address these chronic challenges.
- Create a comprehensive human resources policy for all drug distribution teams (or improve it, if one exists), including clear and realistic job descriptions, training strategies, compensation and incentives, and procedures for managing relationships with program management.
- Revisit or institute a memorandum of understanding between the international partners and the MSPP to ensure that: the auspices under which the program was created, and is being designed, delivered and evaluated can be clearly communicated to the community; the roles and contributions of international and Haitian partners, and their terms of participation are clearly defined; sources and levels of funding from each partner, their contributions to technical and operational support, and their commitment to the success of the program are clearly articulated and accurate; and the policies, governance structure and accountabilities that have been agreed to by the partners are publicly available.
- Develop a communications strategy for the program that directly addresses the public’s concerns as communicated by the drug distribution teams, such as the rates and severity of side effects, and confusion about the LF MDA program lasting more than five years.
